# Editorial: Multi-Omics Study in Revealing Underlying Pathogenesis of Complex Diseases: A Translational Perspective

**DOI:** 10.3389/fgene.2021.789294

**Published:** 2021-10-22

**Authors:** Yue-Miao Zhang, Yong-Fei Wang, Humaira Rasheed, Jurg Ott

**Affiliations:** ^1^ Research Units of Diagnosis and Treatment of Immune-Mediated Kidney Diseases, Renal Pathology Center, Key Laboratory of Renal Disease, Key Laboratory of CKD Prevention and Treatment, Renal Division, Department of Medicine, Peking University First Hospital, Institute of Nephrology, Peking University, Ministry of Health of China, Ministry of Education of China, Chinese Academy of Medical Sciences, Beijing, China; ^2^ State Key Laboratory of Genetic Resources and Evolution, Kunming Institute of Zoology, Chinese Academy of Sciences, Kunming, China; ^3^ Department of Paediatrics and Adolescent Medicine, The University of Hong Kong, Hong Kong, SAR China; ^4^ K.G. Jebsen Center for Genetic Epidemiology, Department of Public Health and Nursing, NTNU, Norwegian University of Science and Technology, Trondheim, Norway; ^5^ MRC Integrative Epidemiology Unit (IEU), Bristol Medical School, University of Bristol, Bristol, United Kingdom; ^6^ Faculty of Medicine, University of Oslo, Oslo, Norway; ^7^ Laboratory of Statistical Genetics, Rockefeller University, New York, NY, United States

**Keywords:** multi-omics study, bioinformatics, translational perspective, disease pathogenesis, clinical implications

**Editorial on the Research Topic**

**Multi-Omics Study in Revealing Underlying Pathogenesis of Complex Diseases: A Translational Perspective**

Genetics contributes to our understanding of the development of complex diseases. In the past few years, a large number of disease-associated loci have been identified using genome-wide association studies (GWASs). However, the causal genes underlying the associated loci remain unclear and how to translate these findings into clinical utility is the main challenge in the post-GWAS era. In recent years, genetic association studies were extended to identify quantitative trait loci (QTL) for intermediate molecular traits using transcriptomic, epigenetic, proteomic, metabolomic, and microbiomic data. The growing availability of vast resources of these omics data combined with well-developed methodologies provide a rapid and cost-effective approach to prioritize the most likely causal genes. These genes can, in turn, help us elucidate disease pathogenesis and prioritize drug targets.

How to harmonize and integrate these multi-omics data is the first question we have to face. In this research topic, we wish to provide examples of studies in integrating multi-omics data sets to discover disease pathogenesis and develop new treatments from a translational perspective. The methods used can be roughly divided into three classes: causal inference methods (i.e., Mendelian Randomization-MR), variant colocalization methods (i.e., COLOC) and co-expression methods (i.e., weighted gene co-expression network analysis-WGCNA). Specifically, MR is a statistical approach widely used in biomedical sciences, which uses genetic variants, typically single nucleotide polymorphisms (SNPs), as a natural experiment to estimate the causal effects of a risk factor on an outcome (Davies et al., 2018). Colocalization methods consider the GWAS and QTLs summary statistics at a locus jointly and probabilistically test if the two signals are likely to be generated by the same causal variant (Giambartolomei et al., 2014). WGCNA combines individual expression datasets of diseases together, and then clusters and screens the shared gene modules between them, which is then used for downstream network analyses (Langfelder and Horvath, 2008).

Adriaan et al. demonstrated how to prioritize the causal genes involved in disease pathogenesis by integrating the approaches of MR, COLOC, LD overlap and DEPICT. Using these methods, they identified 118 prioritized genes across 50 Celiac disease-associated regions. Fifty-one of these genes are targets of approved drugs. The Celiac disease-prioritized genes affect expression of the 172 genes in trans. Among them, the tumor necrosis factor receptor-associated factor-type zinc finger domain containing protein 1 (TRAFD1) is a master regulator of trans-effects, implicating the role of IFNγ signaling as well as with MHC I antigen processing in pathogenesis of Celiac disease. Wang et al. generated a high-resolution contact map from epithelial ovarian cancer (EOC) cells with two H3K27ac-HiChIP libraries and analyzed the underlying gene networks for 15 risk loci identified from the largest EOC GWAS.

Multiple omics data can also help understand shared and/or distinct mechanisms between diseases. IgA nephropathy (IgAN) and lupus nephritis (LN) are the most common forms of nephritis. However, the differences and similarities at molecular levels haven’t been investigated. Jia et al. used gene expression datasets as a quantitative readout of peripheral blood mononuclear cell (PBMC)- and kidney-based molecular phenotypes to analyze the similarities and differences between the two forms of nephritis. They found a significant positive correlation with the kidney expression profiles of IgAN and LN samples, but no significant correlation with PBMC. Using weighted gene co-expression network analysis, they identified some shared pathways, including immunological pathways involved in the glomerulopathy, and cell death and extracellular vesicle pathways involved in the tubular injury.

In addition, drug responses can be varied among patients. Clopidogrel resistance (CR), which attenuates individual responses to clopidogrel therapy, is suggested to be the main reason for recurrent cardiovascular events. Previously, genetic variants in cytochrome P450 isoenzymes (CYPs), such as CYP2C19*2 and CYP2C19*3, have been identified to be associated with CR. Beyond genetics, Yang et al. compared the complete genomic methylation patterns of blood samples from patients with CR and non-CR. They identified significantly different patterns of DNA methylation at 7,098 sites, and four of them, including cg23371584, cg15971518, cg04481923 and cg22507406, were validated independently. Liu et al. interpreted the effects of sodium glucose cotransporter 2 (SGLT2) inhibitors based on their genetic findings, offering a promising strategy to predict the success of drug discovery. Drug targeting human proteins will result in potential on-target benefits but could also exhibit unintended effects (adverse effects). Thus, the spatial distribution of drug targets would help predict the potential on-target effects as well as adverse effects. SLC5A2 is the drug target for SGLT2 inhibitors. It is specifically expressed in kidney tubules but not other tissues, supporting the relatively less adverse effects of SGLT2 inhibitors compared to placebo (Perkovic et al., 2019). Additionally, no significant expression QTLs of SLC5A2 in tubulointerstitial compartments have been observed, suggesting that the genetic variants are less likely to affect gene expression of human SLC5A2 in kidney tubules. This is consistent with the potential wide benefits of SGLT2 inhibitors across populations.

Finally,Ji et al. noted that the SNPs identified by GWAS account for only a small proportion of the heritability, which is likely due to disease complexity and environmental factors. For example, hypertension is among the first traits fully investigated using GWAS. Currently, more and more studies focus on individual quantitative traits, including systolic blood pressure, diastolic blood pressure, and pulse pressure, as was done early in rate models (Vincent et al., 1997). These studies may help us better understand the mechanisms for disease development.
